# Cancer Incidence Among People With a Prior Hospital Record of Depression in Scotland, 1991–2019: A Cohort Study

**DOI:** 10.1002/cam4.70496

**Published:** 2025-01-08

**Authors:** Thulani Ashcroft, Kelly Fleetwood, Christine Campbell, Caroline A. Jackson

**Affiliations:** ^1^ Usher Institute, Usher Building The University of Edinburgh Edinburgh UK

**Keywords:** cancer, cohort, depression, incidence, oncology

## Abstract

**Objective:**

Current evidence on the association between depression and cancer risk is conflicting, with little understanding of how associations vary by time period or sociodemographic factors. We aimed to compare cancer incidence in people with versus without a previous hospital admission record for depression, by sociodemographic factors and over time.

**Methods:**

We conducted a cohort study using national linked data in Scotland from 1991 to 2019. We calculated sex‐stratified age standardised incidence rates for all cancers, lung, female breast, colorectal and prostate cancer, and used quasi‐Poisson regression models to obtain sex‐specific estimates of cancer incidence and relative risks of cancer in those with versus without a prior hospital admission record of depression.

**Results:**

There were 128,654 people with a hospital record of depression with 12,802 incident cancers and 847,656 cancers among those without depression. Age‐standardised cancer incidence rates were higher in both males and females with versus without depression. Depression was associated with a 20%–30% increased risk of all cancers combined, a difference that did not vary by sex, age or deprivation and persisted over three decades. Depression was associated with higher risks of lung (RR 1.79, 95% CI 1.70–1.88) and colorectal cancer (RR 1.12, 95% CI 1.05–1.19), but not breast or prostate cancer.

**Conclusions:**

We identified an entrenched disparity in cancer incidence by depression status. Further research should identify underlying mechanisms and inform cancer prevention strategies in this vulnerable group. Meanwhile, health care professionals have a key role to play in optimising physical health care for people with depression.

## Graphical abstract

Existing evidence on depression and cancer risk is conflicting, likely reflecting differences in methodology, as well as study limitations, such as small study population and short follow‐up periods. Moreover, there has been almost no investigation of how any mental health disparity in cancer risk has changed over time or differs by population sub‐group. We therefore addressed these gaps in a cohort study using Scottish linked administrative healthcare data to compare cancer incidence (all cancers and common cancer subtypes) in those with versus without a hospital admission record for depression, over a 29‐year study period.

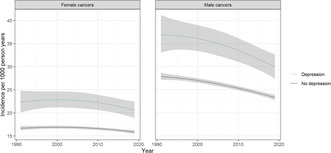

## 1 Background

People with mental illness, including depression, have a shorter life expectancy and poorer health outcomes than the general population [1]. The majority of research in this area has focused on cardiometabolic disease with comparatively less investigation of the risks and outcomes of cancer [1]. Studies on cancer incidence in those with prior depression have produced mixed results, leading to uncertainty as to the true association between depression and cancer incidence. Systematic reviews have combined depression with anxiety [2] and stress [3] and those on depression alone have produced conflicting results. Some report that depression is associated with a higher risk of all cancers combined and lung cancer [4, 5], while others reported no association between depression and all cancers, lung and breast cancer [6, 7] and a recent meta‐analysis only found an association with lung cancer [8]. There is high heterogeneity between studies in terms of study design, definition of depression, number of participants, length of study period and adjustment for confounders.

Even among cohort studies, findings are inconsistent with various limitations, such as inclusion of selected and potentially less representative study populations [9, 10], small study sizes that may have reduced statistical power to detect statistically significant differences in cancer incidence [11, 12] and potential reverse causality due to short follow‐up period [13]. Some studies have examined the association between depression and cancer by age and sex [13, 14] but none have looked at whether the association between depression and cancer incidence has changed over time or if there is a difference by socio‐economic status. Understanding this is critical for assessing whether mental illness disparities in cancer incidence have changed over time (which has implications for population health policy and practice) and informing targeted cancer prevention strategies, the success of which may vary between population sub‐groups.

To address these gaps in the literature, in the present study, we aimed to investigate cancer incidence in Scotland in those with versus without a prior hospital admission record of depression, by time period and sociodemographic factors. Our secondary objective was to compare the association between depression and incidence of the four commonest cancers in Scotland—lung, female breast, colorectal and prostate cancer.

## 2 Methods

We wrote this article in accordance with the Reporting of studies Conducted using Observational Routinely‐collected Data (RECORD) guidance [15].

### 2.1 Study Design, Setting and Population

Each individual who accesses health care in Scotland is given a unique community health index number. This can be used to link records from multiple routinely collected health datasets, including Scottish Morbidity Records (SMR) such as the admission and day case records from acute (general) hospitals and psychiatric hospitals. We received pseudonymised individual level health data from NHS Public Health Scotland, for people with a diagnosis of depression. This included SMR records, and linked death and Scottish national cancer registry records. For the whole Scottish population, we received aggregated data on cancer incidence by age group, sex, calendar year and area‐based deprivation quintile. Information on mid‐year population estimates by age group, sex and deprivation quintile were provided by National Records of Scotland. Using these data, we created a nationwide retrospective dynamic cohort [16, 17, 18] study from 1 January 1991 to 31 December 2019. As described below, this approach allowed us to compare, for each year of the study period, cancer incidence in those with a prior hospital record of depression to cancer incidence in the general population who did not have a prior hospital record of depression, stratifying by sociodemographic factors.

### 2.2 Depression and Comparison Groups

We defined depression as a hospital admission record of depression at age 18 years or older between 1 January 1981 and 31 December 2019. We only used admission records, and not hospital outpatient records, because the latter does not routinely record diagnoses. We identified depression using International Classification of Diseases (ICD) versions 9 (296.1, 298.0, 300.4, 311) and 10 codes (F32‐F33).

Among people with a hospital admission record of depression, we identified incident cancer diagnosed in adults aged 40 years or older between 1 January 1991 and 31 December 2019, via linkage to the cancer registry. We excluded people with cancer diagnosed at age less than 40 years due to the relatively rare occurrence of cancer at younger ages, and people diagnosed with cancer prior to the date of hospital admission record of depression. For data from 1996 onwards, we used ICD‐10 codes to identify all malignant cancers (ICD10 C00‐C97), lung cancer (C34), breast cancer (females only; C50), colorectal cancer (C50) and prostate cancer (C61). For pre–1996 data, we used ICD‐9 codes mapped to corresponding ICD‐10 codes (Table S1). We defined incident cancer as those with no prior record of malignant cancer in the 10 years preceding the cancer diagnosis, effectively applying a 10‐year ‘look‐back period’.

To estimate the number of cancer events for the comparison group of people without a prior hospital admission record of depression, we subtracted the number of incident cancer events among the depression group from the total number of cancer events in the general population, stratifying by year, age, sex and deprivation quintile (at time of cancer diagnosis).

For the depression group, we estimated individual person‐time‐at‐risk as the number of days from study entry (1 January 1991 or date of first hospital admission record of depression if this occurred during the study period) to the earliest of: date of first cancer diagnosis, date of death or end of study period (31 December 2019). We totalled the number of cancer events and person‐time‐at‐risk stratified by age group, year, sex and deprivation quintile. For the comparison group, we estimated person‐years‐at‐risk by subtracting the person‐years‐at‐risk for those with depression from the mid‐year general population estimates (provided by National Records Scotland), stratified by year, age, sex and area‐based deprivation quintile.

### 2.3 Sociodemographic Factors

The age at cancer occurrence was obtained from cancer registry data (either as individual or aggregated data). We used age as a categorical variable in 5‐year age bands to be consistent with the available aggregated cancer counts and mid‐year general population estimates. Sex was provided as individual level data for those with an admission for depression and aggregate data for those without.

In Scotland, area‐based deprivation is measured using the Carstairs Index and the Scottish Index of Multiple Deprivation (SIMD). For this study, we used the Carstairs Index, which is recommended for analyses that include pre–1996 data [19]. The Carstairs Index is a relative measure of material deprivation for each postcode sector, based on four standardised census variables: car ownership, low occupational social class, overcrowding and male unemployment. We used deprivation at point of cancer diagnosis when categorising cancer counts by deprivation in the groups with and without depression. We used deprivation at time of earliest hospital admission record for depression when creating person‐years of follow‐up for the group with depression.

### 2.4 Statistical Analyses

We calculated age standardised incidence rates per 1000 person years using the European Standard Population 2013, for all cancers and the four site‐specific cancers (lung, female breast, colorectal and prostate) by time period and sex. The first time period was 4 years (1991–1994) and subsequent periods were 5 years.

We modelled incidence of all cancers and site‐specific cancers using quasi‐Poisson regression models, from which we estimated rate ratios with 95% confidence intervals (CIs) and the predicted incidence of all cancers per 1000 person years. We have referred to rate ratios hereafter as relative risks (RRs) for ease of understanding. Quasi‐Poisson models were required to allow for overdispersion [20]. Age group, sex and deprivation quintile were included as categorical variables, while year was included as a continuous variable with both linear and quadratic terms. Modelling for all cancers included two‐way interaction terms between each variable as they improved the fit of the model. All interactions terms included were tested for significance using the *F*‐test. Findings from the quasi‐Poisson model were illustrated for people aged 70–74 years (the age group which included the mean age of cancer onset in the depressed group) from the middle deprivation quintile and, where appropriate, the most recent year (2019). To address the secondary objective of our study, we also analysed site‐specific cancers using quasi‐Poisson models adjusted for age group, sex, and deprivation quintile, but without the inclusion of interaction terms.

Since we identified depression recorded in either general or psychiatric hospital admission records, the exposed group may have been heterogeneous in terms of depression severity. To explore whether findings were consistent when restricting analyses to those with likely severe depression only, we performed sensitivity analysis defining depression based on psychiatric hospital admission records only. We conducted all analyses using R software version 4.0.5 [21]. Interactive figures based on quasi‐Poisson models showing predicted incidences for all ages, years and deprivation quintiles are available online (https://ashcroft.shinyapps.io/CancerIncidenceShinyApp/).

## 3 Results

### 3.1 Descriptive Findings

**FIGURE 1:**
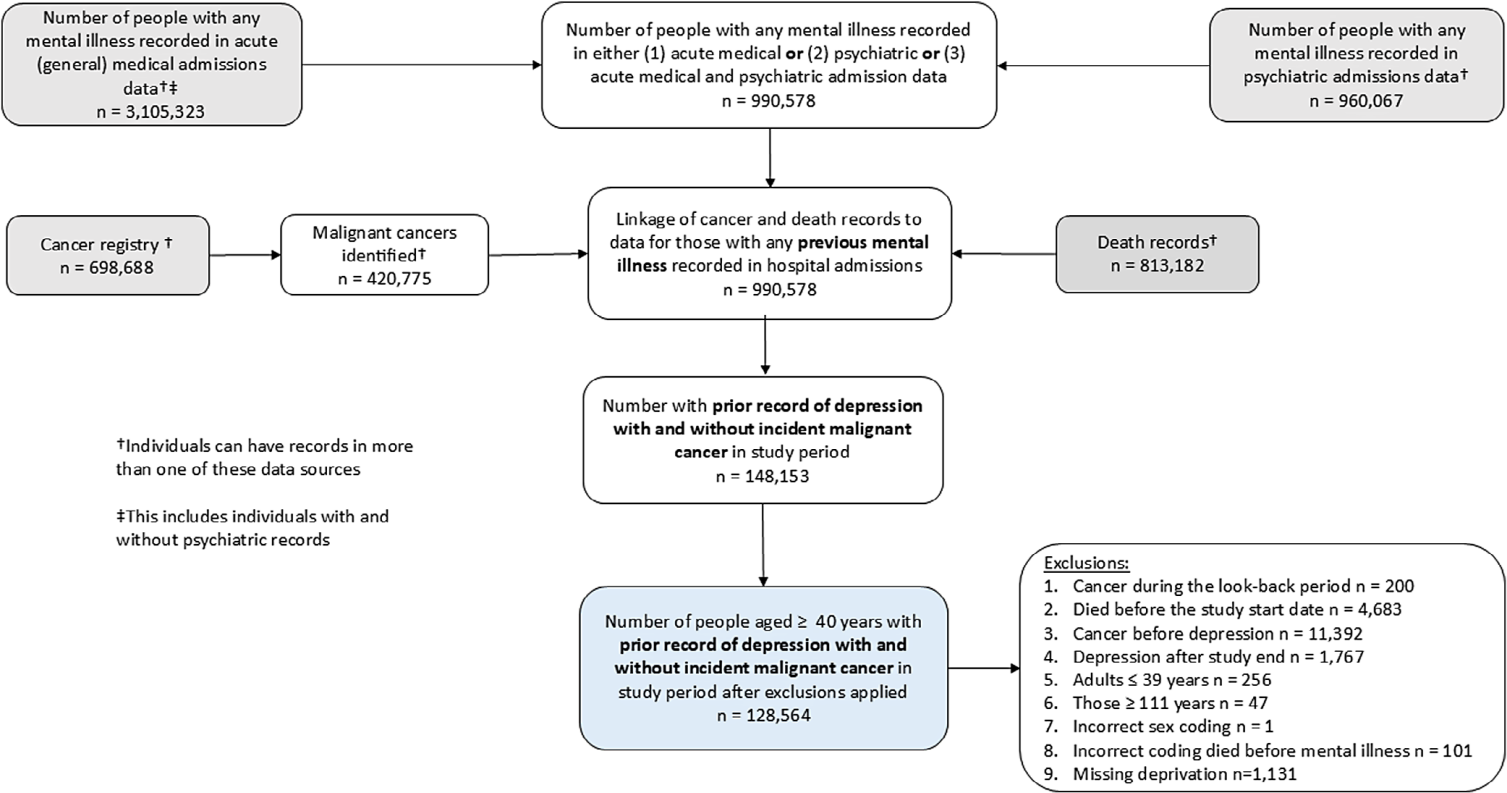
Flow chart showing creation of the group of individuals with prior hospital admission for depression using Scottish health records.

We identified 148,153 people with a hospital admission record for depression, 128,654 of whom met our inclusion criteria (see Figure 1 for creation of group with prior depression). Of these, 64% were female, with the mean age at earliest hospital admission record of depression slightly higher in women (54.9 years (± 20.6 SD)) than men (52.5 (± 19.0 SD); Table S2). Among those with depression there were 12,802 malignant cancers (men = 4690; women = 8112). Mean age of cancer onset was 70.0 (± 11.6 SD) years and 69.8 (± 13.2 SD) years for men and women respectively. Of the site‐specific cancers, lung cancer was the most frequent (*N* = 2461; Table S3), followed by breast cancer in females (*N* = 1485), colorectal cancer (*N* = 1139) and prostate cancer (*N* = 638).

As an indication of the general population size for Scotland during the study period, mid‐year population estimates for those 40 years and over, ranged from 2,163,126 in 1991 to 2,822,637 in 2019. Over the whole study period the number of cancers among those with no prior hospital admission record for depression was 847,656, with a similar proportion in men and women. As was the case for people with depression, lung cancer was the commonest (*N* = 115,585), then breast (*N* = 95,485) colorectal (*N* = 85,458) and prostate (*N* = 66,716).

### 3.2 Age‐Standardised Incidence Rates for All Cancers and Site‐Specific Cancers

**FIGURE 2:**
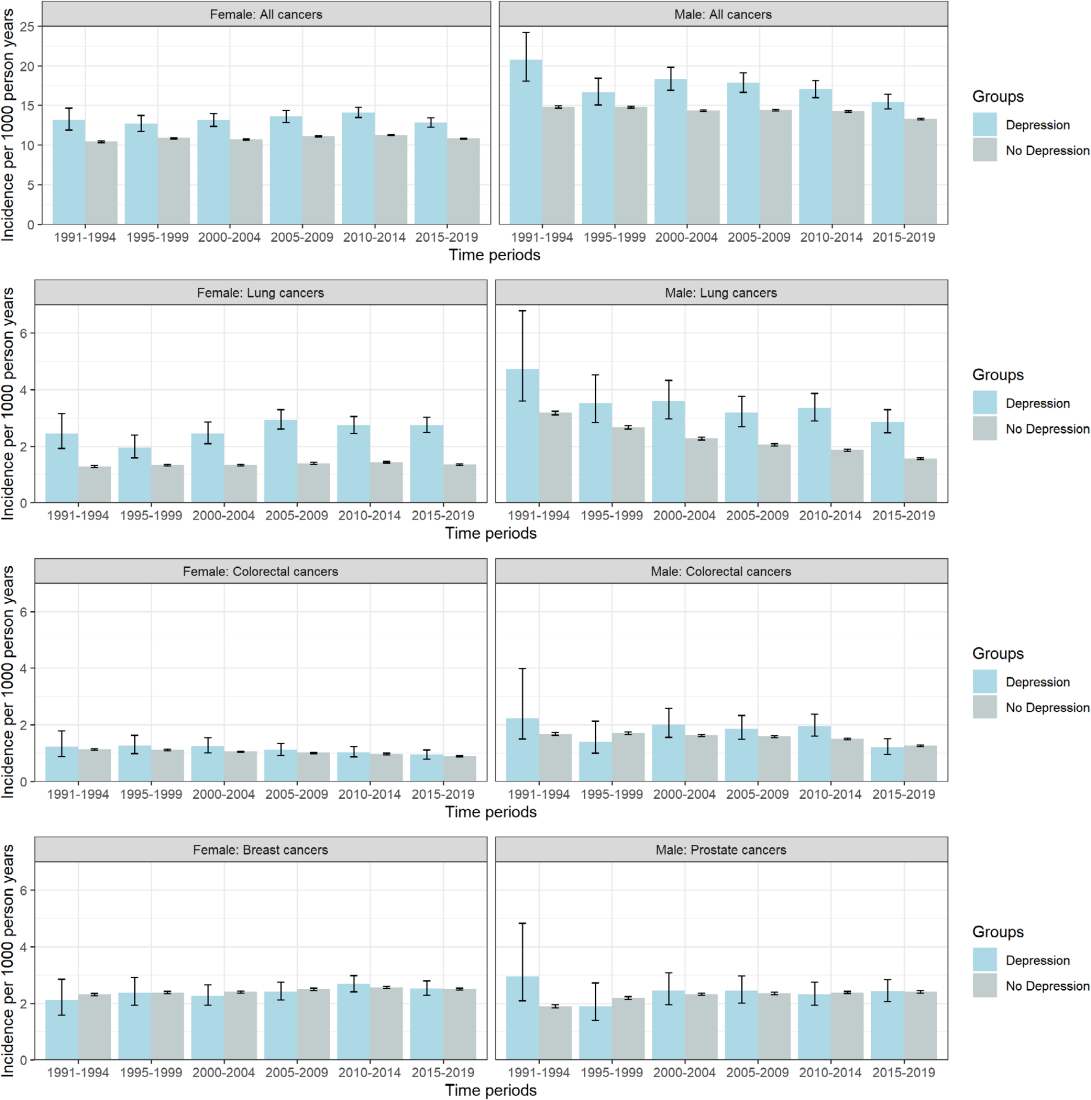
Age‐standardised incidence rates† of all cancers and site‐specific cancers in those with and without a previous hospital admission record of depression, stratified by sex, by time‡, in Scotland, 1991–2019. †Standardised using European Standard Population 2013; ‡5 year periods apart from 1991 to 1994 a 4‐year period.

Age standardised incidence rates for all cancers and lung cancer were higher for people with versus without depression from 1991 to 2019. For females, in the period 2015–2019, the age‐standardised incidence per 1000 person years of all cancers in those with depression was 12.82 (95% CI 12.26–13.41) versus 10.81 (95% CI 10.73–10.89) in those without. For men in the same period, incidence per 1000 person‐years was 15.44 (95% CI 14.55–16.41) for those with depression and 13.28 (95% CI 13.19–13.37) for those without (see Figure 2, with values reported in Table S4). Rates of colorectal, female breast and prostate cancers were similar for people with and without depression. There was no clear change in the incidence over time for either comparison group for all cancers, colorectal, breast or prostate cancer. In contrast, lung cancer incidence has decreased over the study period in men both with and without a prior diagnosis of depression. Compared to women, men in both comparison groups had a higher incidence of all cancers, lung and colorectal cancer.

### 3.3 Modelling of Incidence of All Cancers

**FIGURE 3:**
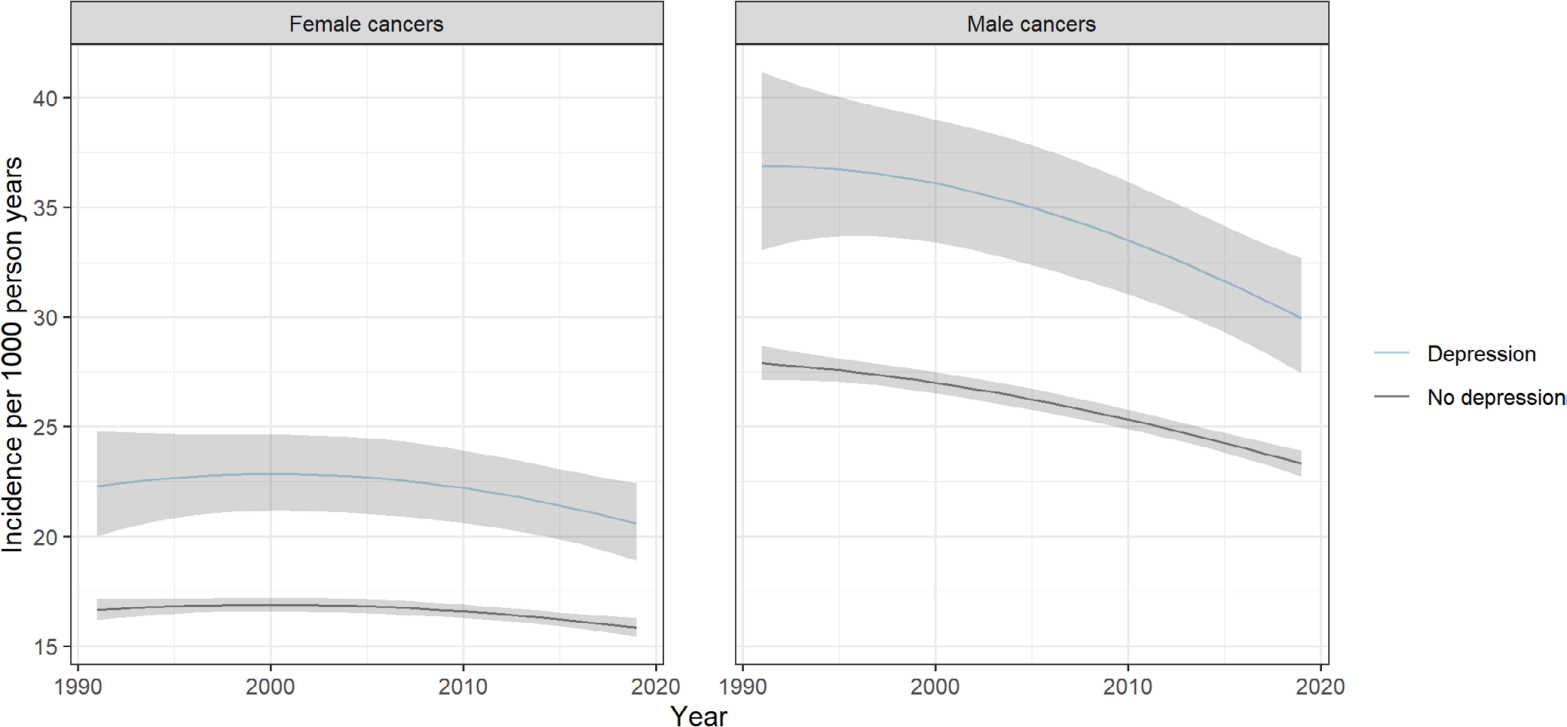
Predicted sex‐stratified incidence of all cancer for those with and without a hospital admission record for depression, aged 70–74 and in the middle deprivation quintile, in Scotland, from 1991 to 2019.

Generally, cancer incidence in males and females was higher in those with versus without depression. Since the model includes interaction terms, the effect estimate for depression varies slightly by sex, age, deprivation quintile and time. Findings were however broadly consistent across these groups. Results for people aged 70–74 years from the middle deprivation quintile are illustrated in Figure 3; results for other age groups and deprivation quintiles are available on the interactive app (https://ashcroft.shinyapps.io/CancerIncidenceShinyApp/). Overall, in this age‐group the excess risk of all cancer was around 20%–30% higher in those with versus without a history of depression, with some variation in point estimates by sex, year and deprivation level. For example, in 2019, cancer incidence was 32% (RR 1.32, 95% CI 1.20–1.44) and 28% (RR 1.28, 95% CI 1.18–1.40) higher in women and men, respectively, among those aged 70–74 years and living in areas classified as the middle deprivation quintile (Table S5).

**FIGURE 4:**
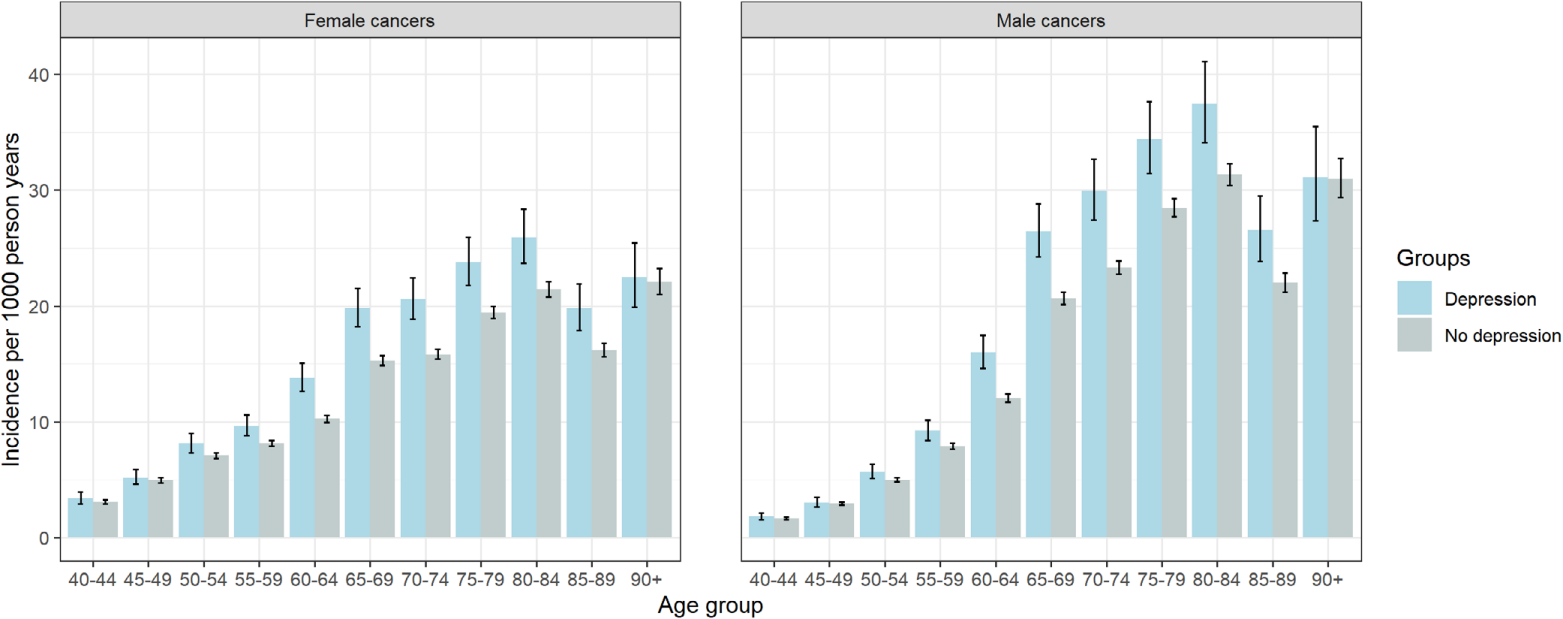
Predicted cancer incidence for those with and without a hospital admission record for depression in the middle deprivation quintile in Scotland in 2019, stratified by sex and age group.

Both people with and without depression had the same pattern of cancer incidence across age groups with peak incidence occurring at either 80–84 years or 85–89 years, depending on calendar year, sex and deprivation quintile (Figure 4 and interactive app). In both groups in all deprivation quintiles, cancer incidence was higher in males than females in age groups above 60 years, whereas cancer incidence was higher in females than males in the youngest age range of 40–54 years.

**FIGURE 5:**
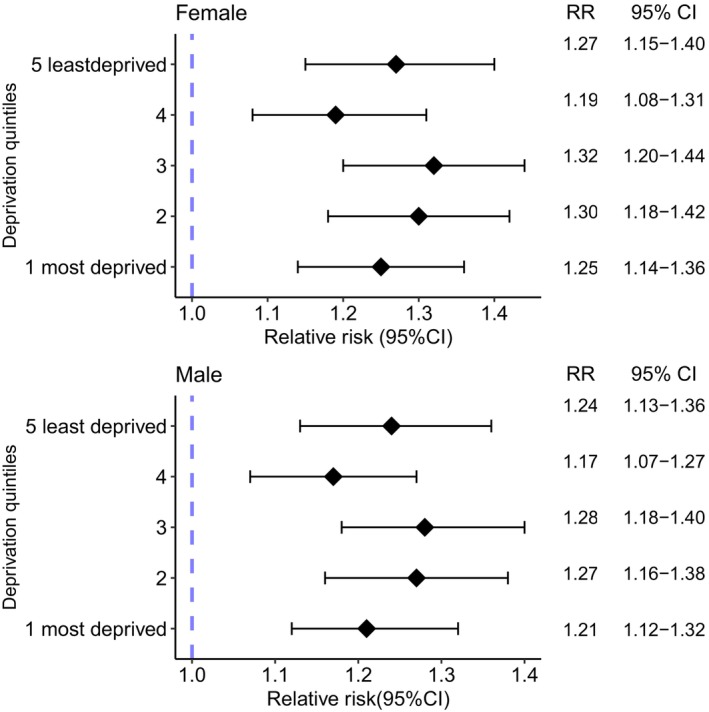
Relative risks (RR) of all cancer for those with versus without a previous hospital admission record for depression, by deprivation quintile in age group 70–74 years, stratified by sex, in Scotland in 2019.

For both people with and without depression, those living in the most deprived areas of Scotland had a higher incidence of cancer than those in the least deprived areas. Within each deprivation quintile, cancer incidence was higher in those with versus without prior depression, but there was no difference in the magnitude of the excess risk of cancer across deprivation quintiles. For example, in 2019, in people aged 70–74 years within the most deprived quintile, depression was associated with a 25% (RR 1.25, 95% CI 1.14–1.36) and 22% (RR 1.21, 95% CI 1.12–1.32) higher risk of cancer in females and males, respectively. In people aged 70–74 years within the least deprived group, the excess risk was similar (females: RR 1.27, 95% CI 1.15–1.40; males: RR 1.24, 95% CI 1.13–1.36; Figure 5 and Table S5).

### 3.4 Modelling of Incidence of Site‐Specific Cancers

Risk of lung cancer was 79% higher (adjusted RR 1.79, 95% CI 1.70–1.88) in those with versus without prior depression. Risk of colorectal cancer was slightly higher in those with versus without depression (RR 1.12, 95% CI 1.05–1.19), but there was no association for female breast (RR 1.00, 95% CI 0.94–1.06) or prostate cancer (RR 1.01, 95% CI 0.93–1.17; Table S6).

### 3.5 Sensitivity Analysis

Among people defined as having depression, almost two‐thirds (92,437 [72%]) had a psychiatric hospital admission record of depression, whereas one‐third only had a general hospital admission record of depression. Defining depression based on psychiatric hospital admission record alone led to the inclusion of 12,011 people with depression and incident cancer during follow‐up, with age at cancer diagnosis (mean 67.3 years ± 12.0 SD) slightly lower than the primary analysis (mean 69.5 ± 12.7 SD). The pattern of findings was very similar to the primary analysis, with age‐standardised incidence slightly higher and rate ratio estimates slightly larger in the sensitivity analyses (Tables S7–S9).

## 4 Discussion

### 4.1 Main Findings

Our analyses using Scottish national data show that incidence of all cancers combined was between 20% and 30% higher in people with versus without a prior hospital admission record for depression, irrespective of age‐group, sex or deprivation level. This disparity was relatively stable over the 29‐year study period. The risk of lung cancer was markedly higher in those with versus without depression. There was also a slight excess risk of colorectal cancer in people with depression, but no difference in risk of female breast or prostate cancer.

### 4.2 Interpretation in Context of Previous Studies

Although systematic reviews on this topic are limited by between‐study heterogeneity, two reviews for depression alone similarly found an increased risk of all cancers, and lung cancer specifically [4, 5]. As in our study, both reviews found that risk of all cancer was 20%–30% higher in those with, compared to those without, depression. However, the excess risks of lung cancer were lower and the confidence intervals far wider than in our study. Retrospective cohort studies using nationwide routine health data have also found results closely aligned to ours reporting that depression was associated with an increased risk of all cancers, lung, and colorectal cancer [13, 14, 22]. The most recent study, used German health administrative data from 2015 to 2018, creating comparison groups of 117,702 individuals each by 1:1 matching of those given ICD‐10 codes for depression and those without codes [13]. However, the largest study, linking broad categories of mental illness and medical conditions (*n* = 5,940,299) using nationwide psychiatric Danish data from 1969 to 2016, reported hazards ratios only slightly above the null for mood disorders combined and all cancers (hazard's ratio 1.08, 95% CI 1.06–1.09) [23]. A small study of only 1319 incident cancer events among those with depression also found no association with all cancer but found increased risk of lung cancer (incident rate ratio 1.36, 95% CI 1.12–1.48) [12].

In contrast recent prospective cohort studies using questionnaires to ascertain depression have not found an association with all cancers. A recent large (*n* = 319,613) meta‐analysis included individual level participant data from multiple prospective cohorts from the Netherlands, UK and Canada to examine the association between depression and cancer incidence. These studies ascertained current depression largely through self‐reported symptoms, with only a few (generally small) studies with depression ascertained by clinical interview [8]. The authors noted that there were missing data (extent of this not reported on) and hence performed a complete case analysis, reporting no association between depression and all cancer risk, in minimally adjusted models, but did find an association between depression and lung cancer risk. Similarly, longitudinal evidence from the HUNT study in Norway found depressive symptomology was associated with an increased risk of lung cancer but not all cancers combined [24].

One key difference which may partly account for divergent results is how depression is measured. Studies using validated questionnaires or clinical interviews may be recording current depressive symptoms and those using health records identifying those with a history of persisting symptoms. Moreover, if the association between depression and cancer is dose‐dependent, these conflicting findings may reflect greater ascertainment of less severe depression in studies assessing short term depressive symptoms. This warrants further investigation through assessment of depression severity in relation to cancer incidence.

We note that our study is the only one which has shown that the higher incidence of cancer in those with a prior admission for depression versus those without is present across age groups, deprivation levels and both sexes and the first to demonstrate the persistence of this health disparity over three decades.

## 5 Implications

Further research is needed to understand the mechanisms underlying the association between depression and cancer incidence and mortality. These mechanisms will likely vary by cancer subtype, depending on the extent to which, for example, different lifestyle factors play an aetiological role in cancer development. However, while lifestyle factors likely play an important role, future studies should also consider other factors. For instance, given the potential role of chronic inflammation in the aetiology of both disorders [25, 26] it is important to explore the contributory role of inflammation in explaining the observed link between depression and cancer risk. Future research could, for example, utilise large‐scale biomedical research resources such as the UK Biobank [27] and 45 And Up study [28], which benefit from their incredible depth and breadth of data on participants, with linkage to multiple routine data sources as well as collection of biomarker data.

Unfortunately, we were unable to examine depression in relation to stage of cancer at diagnosis in the present study. This is however an important area of research, given the increased risk of cancer in people with depression and also that people with mental illness may experience delayed cancer diagnosis. Studies on the latter have various methodological limitations which limit interpretation of existing evidence [29]. However, evidence from the most methodologically robust studies suggest that compared to those without mental illness, people with a pre‐existing illness have a higher risk of advanced disease at cancer diagnosis [29]. Analysis of pre–COVID‐19 data in a Danish cohort found those with mental illness had a 40% higher risk of presenting as an unplanned admission for cancer than those without pre‐existing mental illness [30]. Differences in how people with pre‐existing depression navigate cancer pathways compared to those without mental illness can influence presentation to health services and attendance at cancer screening [31, 32]. Differential access to cancer screening programmes, where available, could partly explain negative or null associations between depression and incidence of individual cancer type. Implementation of lung cancer screening in coming years has the potential to widen health inequalities for those with depression with anticipated increased participation (and hence improved survival) among those without mental illness, but also brings opportunities for targeted interventions ideally through inter‐disciplinary working.

The Lancet Psychiatry Commission [1] highlighted the physical health disparities for people living with mental illness and the need to integrate physical and mental health care. Future research should focus on investigating how depression is associated with receipt of cancer care across the entire patient pathway, from prevention (including screening) through to palliative care. This may be through being alert to the increased diagnostic difficulty due to diagnostic overshadowing [33] and providing guidance and education for people with depression. Our study has shown that the gap in cancer incidence between those with and without a hospital admission record for depression has not improved over time and is consistent across sociodemographic factors, highlighting the need for renewed efforts to improve cancer prevention in this at‐risk sub‐population. Given our study period preceded the COVID‐19 pandemic and there have since been significant effects on health services, it will be imperative to examine whether the pandemic has exacerbated these disparities.

## 6 Limitations

Our study does have some limitations. By including only people with a general or psychiatric hospital admission record for depression, we likely included people with more severe depression. In a sensitivity analysis where we based our depression definition only on psychiatric hospital admission records we found very similar results to the primary analyses. If there is a dose–response relationship between depression and cancer risk, then our findings may not necessarily be generalisable to people with less severe depression. Conversely, if depression is associated with cancer risk irrespective of depression severity, we may have underestimated the association between depression and cancer risk. However, by using national hospital data we were able to include a highly representative study population and use objective measures for both depression and cancer. Secondly, selection bias may have occurred as over 11,000 people with a date of cancer ascertainment before an admission record of depression were excluded. This exclusion was to minimise the risk of reverse causation, although we did not exclude cancers diagnosed within a year of hospital admission for depression and so some residual reverse causation may exist. Since cancers tend to have a long latency period and our study period was 29 years, this enabled us to identify a large number of cancers as well as examine changes over time and the effect of interactions by sociodemographic factors.

Limitations could have occurred with the comparison group which included people with a history of psychiatric disorders other than depression, which may have diluted observed associations. We also used mid‐year population estimates and so this group would have included those with prior cancer who may have either undiagnosed depression or depression diagnosed in outpatient clinic or primary care. Errors caused by the use of mid‐year population estimates are expected to be small as the incidence of both depression and cancer is low relative to the size of the population.

We did not have any information on lifestyle factors and so could not examine the effect of adjusting for these on the observed associations. Previous studies found that such adjustment attenuated the associations seen in minimally adjusted models [8]. However, where the focus is to identify, rather than explain, disparities, this potentially leads to overadjustment, through adjusting for factors that may lie on the causal pathway between depression and cancer. The purpose of our study was to investigate the association between depression and cancer risk and how this varied by time and sociodemographic factors, rather than to seek to explain possible mechanisms underlying associations.

## 7 Conclusions

Our study has demonstrated an entrenched disparity in cancer incidence between people with and without a previous hospital admission record for depression in Scotland. These disparities were present to a similar degree by sex, age‐group and area‐based deprivation level and were evident for all cancers, lung, and colorectal cancer. Future research should focus on developing and evaluating improved cancer prevention initiatives and investigating cancer screening and stage of cancer at diagnosis by depression status. Identifying and mitigating health system factors (pathway and professional) that contribute to later presentation or delayed diagnosis in this vulnerable population will also be important. Healthcare professionals providing care for those with depression should be aware of this excess risk; improved shared decision‐making may improve the overall physical health outcomes for this group.

## Author Contributions

**Thulani Ashcroft:** conceptualization (equal), data curation (lead), formal analysis (lead), investigation (lead), methodology (lead), resources (lead), software (lead), visualization (lead), writing – original draft (lead). **Kelly Fleetwood:** conceptualization (supporting), data curation (supporting), formal analysis (supporting), methodology (supporting), supervision (supporting), writing – review and editing (supporting). **Christine Campbell:** conceptualization (supporting), supervision (supporting), writing – review and editing (supporting). **Caroline A. Jackson:** conceptualization (supporting), data curation (supporting), formal analysis (supporting), methodology (supporting), resources (supporting), software (supporting), supervision (supporting), writing – original draft (supporting), writing – review and editing (supporting).

## Ethics Statement

Permission to conduct this study with pseudonymised, non‐consented data was obtained from the Public Benefit and Privacy Panel of National Health Service National Services Scotland, reference number 1516‐0626.

## Consent

The authors have nothing to report.

## Conflicts of Interest

The authors declare no conflicts of interest.

## Supporting information


Table S1.

## Data Availability

The data that support the findings of this study is available from the Electronic Data Research and Innovation Service of National Services Scotland. Restrictions apply to the availability of these data, which were used under licence for this study. However, data are available upon reasonable request from the authors with the permission of the Public Benefit and Privacy Panel of National Health Service, National Services Scotland.
