# Supplementary material for: Cancer Incidence Among People With a Prior Hospital Record of Depression in Scotland, 1991–2019: A Cohort Study
Source: Cancer Med. 2025 Jan 8;14(1):e70496. doi: 10.1002/cam4.70496 (PMC11711213; doi:10.1002/cam4.70496)
Supplement: Supplementary file 1 — Table S1. [file CAM4-14-e70496-s001.docx]

# Supplementary materials

## Supplementary Table 1: ICD 10 codes for cancers mapped to ICD 9 codes

| **Cancers** | **ICD 9 codes** | **ICD 10 codes** |
| --- | --- | --- |
| All malignant cancers | 140-208; 2386; 2362 | C00-96 |
| Lung cancer | 162.2-162.9 | C34 |
| Female Breast cancer | 174 | C50 |
| Colorectal cancer | 153.0-154.1 | C18-C20 |
| Prostate cancer | 185 | C61 |

## Supplementary Table 2: Characteristics of people with a hospital admission record of depression, with and without incident cancer, in Scotland, 1991-2019

|  | Hospital admission record of depression | Male | Female |
| --- | --- | --- | --- |
| Total number with depression | 128,564 | 46,337 | 82,227 |
| Mean age in years (SD) of depression admission | 54.1 (20.0) | 52.5 (19.0) | 54.9 (20.6) |
| Total number of cancer events | 12802.0 | 4690.0 | 8112.0 |
| Mean age in years (SD) at cancer diagnosis |  |  |  |
| All cancer | 69.5 (12.7) | 70.0 (11.6) | 69.8 (13.2) |
| Lung | 69.0 (10.7) | 68.9 (10.4) | 69.1 (10.8) |
| Breast | NA | NA | 65.4(13.9) |
| Colorectal | 72.4 (12.0) | 70.4 (11.8) | 73.7 (12.0) |
| Prostate | NA | 71.2 (10.0) | NA |

## Supplementary Table 3: Frequency of all cancer and site-specific cancer diagnoses, by history of hospital admission record for depression, sex, and deprivation

|  | Number (%) of cancers in those with depression | | | Number (%) of cancers in those without depression | | |
| --- | --- | --- | --- | --- | --- | --- |
|  | All | Male | Female | All | Male | Female |
| **All cancers** | **12802.0** | **4690.0** | **8112.0** | **847656** | **422369** | **425287** |
| 1 (Most) | 3130 (24.2) | 1127 (24.0) | 2003 (24.7) | 168356 (19.9) | 83629 (19.8) | 84727 (19.9) |
| 2 | 2735 (21.4) | 971 (20.7) | 1764(21.7) | 163343 (19.3) | 81050 (19.2) | 82293 (19.3) |
| 3 | 2689 (21.0) | 987 (21.0) | 1702 (21.0) | 168699 (19.9) | 83843 (19.9) | 84856 (20.0) |
| 4 | 2330 (18.2) | 890 (19.0) | 1440 (17.8) | 175139 (20.7) | 87562 (20.7) | 87577 (20.6) |
| 5 (Least) | 1918 (15.0) | 715 (15.2) | 1203 (14.8) | 172119 (20.3) | 86285 (20.4) | 85834 (20.2) |
| **Lung** | **2461.0** | **914.0** | **1547.0** | **115585.0** | **63244.0** | **52341.0** |
| 1 (Most) | 760 (30.9) | 283 (31.0) | 477 (30.8) | 32493 (28.1) | 17658 (27.9) | 14835 (28.3) |
| 2 | 605 (24.6) | 224 (24.5) | 381 (24.6) | 25403 (22.0) | 13949 (22.1) | 11454 (21.9) |
| 3 | 457 (18.6) | 168 (18.4) | 289 (18.7) | 22686 (19.6) | 12472 (19.7) | 10214 (19.5) |
| 4 | 372 (15.1) | 134 (14.7) | 238 (15.4) | 19765 (17.1) | 10950 (17.3) | 8815 (16.8) |
| 5 (Least) | 267 (10.8) | 105 (11.5) | 162 (10.5) | 15238 (13.2) | 8215 (13.0) | 7023 (13.4) |
| **Breast** | **N/A** | **N/A** | **1485.0** | **N/A** | **N/A** | **95485.0** |
| 1 (Most) |  |  | 347 (23.4) |  |  | 16717 (17.5) |
| 2 |  |  | 323 (21.8) |  |  | 18039 (18.9) |
| 3 |  |  | 317 (21.3) |  |  | 19138 (20.0) |
| 4 |  |  | 291 (19.6) |  |  | 20332 (21.3) |
| 5 (Least) |  |  | 207 (13.9) |  |  | 21259 (22.3) |
| **Colorectal** | **1139.0** | **459.0** | **680.0** | **85458.0** | **45462.0** | **39996.0** |
| 1 (Most) | 244 (21.4) | 90 (19.6) | 154 (22.6) | 15996 (18.7) | 8684 (19.1) | 7312 (18.3) |
| 2 | 220 (19.3) | 89 (19.4) | 131 (19.3) | 16132 (18.9) | 8670 (19.1) | 7462 (18.7) |
| 3 | 246 (21.6) | 100 (21.8) | 146 (21.5) | 17237 (20.2) | 9197 (20.2) | 8040 (20.1) |
| 4 | 224 (19.7) | 98 (21.4) | 126 (18.5) | 18285 (21.4) | 9634 (21.2) | 8651 (21.6) |
| 5 (Least) | 205 (18.0) | 82 (17.9) | 123 (18.1) | 17808 (20.8) | 9277 (20.4) | 8531 (21.3) |
| **Prostate** | **NA** | **638.0** | **NA** | **NA** | **66716.0** | **NA** |
| 1 (Most) |  | 127 (19.9) |  |  | 10523 (15.8) |  |
| 2 |  | 131 (20.5) |  |  | 11790 (17.7) |  |
| 3 |  | 146 (22.9) |  |  | 13388 (20.1) |  |
| 4 |  | 122 (19.1) |  |  | 14859 (22.3) |  |
| 5 (Least) |  | 112 (17.6) |  |  | 16156 (24.2) |  |

## Supplementary Table 4: Sex stratified age standardized† cancer incidence (rate per 1,000 person years with 95% confidence intervals (CI)), by history of hospital admission record of depression and time period, in Scotland 1991-2019‡

|  | **All cancer rate per 1,000 person-years (95% CI)** | | **Lung cancer rate per 1,000 person-years (95% CI)** | | **Colorectal cancer rate per 1,000 person-years (95% CI)** | | **Breast (female) and prostate (male) cancer rate per 1,000 person-years (95% CI)** | |
| --- | --- | --- | --- | --- | --- | --- | --- | --- |
| **Period (years)** | **With depression** | **Without depression** | **With depression** | **Without depression** | **With depression** | **Without depression** | **With depression** | **Without depression** |
| **Female** |  |  |  |  |  |  | **Breast** |  |
| 1991-1994 | 13.19 (11.90 -14.66) | 10.41 (10.32-10.50) | 2.44 (1.92-3.15) | 1.28 (1.25-1.32) | 1.23 (0.88-1.79) | 1.12 (1.10-1.15) | 2.11 (1.57-2.85) | 2.30 (2.26-2.35) |
| 1995-1999 | 12.69 (11.73 -13.73) | 10.86 (10.77-10.94) | 1.95 (1.59-2.40) | 1.34 (1.31-1.37) | 1.26 (0.98-1.63) | 1.11 (1.08-1.14) | 2.37 (1.94-2.90) | 2.38 (2.34-2.42) |
| 2000-2004 | 13.15 (12.36 -14.01) | 10.72 (10.64-10.80) | 2.45 (2.10-2.86) | 1.33 (1.30-1.36) | 1.25 (1.01-1.55) | 1.05 (1.02-1.08) | 2.26 (1.93-2.65) | 2.41 (2.37-2.44) |
| 2005-2009 | 13.60 (12.89 -14.34) | 11.11 (11.03-11.19) | 2.93 (2.60-3.30) | 1.40 (1.37-1.43) | 1.11 (0.92-1.35) | 1.01 (0.99-1.03) | 2.41 (2.12-2.75) | 2.50 (2.46-2.53) |
| 2010-2014 | 14.11 (13.46 -14.78) | 11.25 (11.17-11.33) | 2.73 (2.45-3.05) | 1.44 (1.41-1.47) | 1.03 (0.86-1.23) | 0.97 (0.95-0.99) | 2.68 (2.40-2.98) | 2.55 (2.52-2.59) |
| 2015-2019 | 12.82 (12.26 -13.41) | 10.81 (10.73-10.89) | 2.74 (2.49-3.03) | 1.35 (1.32-1.38) | 0.94 (0.79-1.12) | 0.89 (0.87-0.91) | 2.52 (2.28-2.79) | 2.50 (2.47-2.54) |
|  |  |  |  |  |  |  |  |  |
| **Male** |  |  |  |  |  |  | **Prostate** |  |
| 1991-1994 | 20.75 (18.09 -24.20) | 14.81 (14.67-14.96) | 4.73 (3.59-6.79) | 3.18 (3.11-3.24) | 2.23 (1.50-4.00) | 1.67 (1.62-1.72) | 2.96 (2.09-4.82) | 1.89 (1.84-1.95) |
| 1995-1999 | 16.64 (15.08 -18.46) | 14.79 (14.67-14.91) | 3.53 (2.83-4.53) | 2.67 (2.62-2.72) | 1.40 (1.00-2.14) | 1.70 (1.66-1.74) | 1.90 (1.40-2.72) | 2.18 (2.14-2.23) |
| 2000-2004 | 18.30 (16.89 -19.83) | 14.35 (14.24-14.47) | 3.58 (2.97-4.32) | 2.27 (2.23-2.32) | 2.00 (1.56-2.58) | 1.62 (1.58-1.66) | 2.45 (1.96-3.09) | 2.32 (2.28-2.37) |
| 2005-2009 | 17.84 (16.65 -19.12) | 14.42 (14.31-14.53) | 3.18 (2.69-3.76) | 2.06 (2.01-2.10) | 1.86 (1.49-2.33) | 1.58 (1.55-1.62) | 2.44 (2.01-2.97) | 2.35 (2.31-2.40) |
| 2010-2014 | 17.05 (16.01 -18.15) | 14.23 (14.13-14.34) | 3.35 (2.90-3.87) | 1.86 (1.82-1.90) | 1.95 (1.60-2.37) | 1.50 (1.46-1.53) | 2.31 (1.94-2.76) | 2.39 (2.35-2.43) |
| 2015-2019 | 15.44 (14.55 -16.41) | 13.28 (13.19-13.37) | 2.86 (2.48-3.30) | 1.57 (1.54-1.60) | 1.20 (0.95-1.52) | 1.25 (1.22-1.28) | 2.42 (2.06-2.84) | 2.41 (2.37-2.45) |

†Using European standard population 2013

‡First time period 4 years (1991-1994), subsequent time periods 5 years

## Supplementary Table 5: Sex-stratified relative risks (95% CI) for all cancer, comparing people with versus without a hospital admission record of depression, aged 70-74 years, in Scotland, 2000, 2005, 2010, 2015 and 2019.

|  | Relative risks with 95% CI in specific years | | | | |
| --- | --- | --- | --- | --- | --- |
| Area based deprivation | **2000** | **2005** | **2010** | **2015** | **2019** |
| FEMALE |  |  |  |  |  |
| 1 (most deprived) | 1.28 (1.19-1.38) | 1.28 (1.19-1.37) | 1.27 (1.18-1.36) | 1.25 (1.16-1.34) | 1.25 (1.14-1.36) |
| 2 | 1.33 (1.24-1.44) | 1.33 (1.23-1.43) | 1.32 (1.23-1.42) | 1.30 (1.21-1.40) | 1.30 (1.18-1.42) |
| 3 | 1.35 (1.26-1.46) | 1.35 (1.25-1.46) | 1.34 (1.24-1.44) | 1.32 (1.23-1.42) | 1.32 (1.20-1.44) |
| 4 | 1.23 (1.14-1.33) | 1.23 (1.14-1.32) | 1.22 (1.13-1.31) | 1.20 (1.11-1.29) | 1.19 (1.08-1.31) |
| 5 (least deprived) | 1.31 (1.21-1.42) | 1.30 (1.20-1.41) | 1.29 (1.19-1.40) | 1.27 (1.18-1.38) | 1.27 (1.15-1.40) |
| MALE |  |  |  |  |  |
| 1 (most deprived) | 1.26 (1.17-1.36) | 1.26 (1.17-1.36) | 1.25 (1.16-1.34) | 1.23 (1.15-1.33) | 1.22 (1.12-1.32) |
| 2 | 1.32 (1.22-1.42) | 1.31 (1.22-1.42) | 1.30 (1.21-1.40) | 1.28 (1.19-1.39) | 1.27 (1.16-1.38) |
| 3 | 1.34 (1.24-1.44) | 1.33 (1.24-1.44) | 1.32 (1.23-1.43) | 1.30 (1.21-1.41) | 1.28 (1.18-1.40) |
| 4 | 1.21 (1.12-1.31) | 1.21 (1.12-1.31) | 1.20 (1.11 -1.30) | 1.18 (1.08-1.28) | 1.17 (1.07-1.27) |
| 5 (least deprived) | 1.29 (1.19-1.40) | 1.29 (1.19-1.40) | 1.28 (1.18-1.38) | 1.26 (1.15-1.36) | 1.24 (1.13-1.30) |

## Supplementary Table 6: Adjusted rate ratios from multivariable quasi-Poisson models for the association of depression and covariates with cancer incidence, for site-specific cancers

|  | Adjusted Rate ratios and 95% confidence intervals | | | |
| --- | --- | --- | --- | --- |
| Covariates† | **Lung cancer** | **Breast cancer** | **Colorectal cancer** | **Prostate cancer** |
| No depression (reference)  Depression | 1.00  1.79 (1.70-1.88) | 1.00  1.00(0.94-1.06) | 1.00  1.12(1.05-1.19) | 1.00  1.01(0.93-1.17) |
| Age group (years)  40-44  45-49  50-55 (reference)  55-59  60-64  65-69  70-74  75-79  80-84  85-89  +90 | 0.18 (0.17-0.2)  0.50(0.47-0.53)  1.00  2.11(2.02-2.21)  3.57 (3.43-3.72)  5.75 (5.52-5.98)  7.72 (7.42-8.03)  8.76 (8.42-9.12)  9.20 (8.82-9.59)  7.31 (6.97-7.67)  5.92(5.55-6.32) | 0.43 (0.42-0.45)  0.68(0.66-0.70)  1.00  1.01(0.98-1.04)  1.13(1.10-1.16)  1.16(1.13-1.19)  1.04(1.00-1.07)  1.10(1.07-1.14)  1.21(1.17-1.25)  1.23(1.19-1.28)  1.40(1.33-1.47) | 0.26 (0.24-0.28)  0.54(0.51-0.57)  1.00  1.70(1.63-1.77)  2.56(2.46-2.66)  3.91(3.77-4.06)  5.00(4.82-5.19)  6.08(5.86-6.31)  7.43(7.15-7.72)  7.25(6.95-7.56)  7.40(7.02-7.81) | 0.04(0.02-0.05)  0.24(0.21-0.28)  1.00  3.03(2.80-3.28)  6.11(5.68-6.58)  10.78(10.04-11.59)  13.67(12.74-14.70)  15.48(14.41-16.65)  16.11(14.95-16.65)  14.02(12.92-15.23)  16.32(14.69-18.12) |
| Sex  Female (reference)  Male | 1.00  1.58 (1.56-1.61) | N/A | 1.00  1.51(1.48-1.53) | N/A |
| Year | 0.99 (0.99-0.99) | 1.00(1.00-1.01) | 0.99(0.99-0.99) | 1.01(1.01-1.01) |
| Area based deprivation  1 (most deprived)  2  3  4  5 (least deprived) (reference) | 2.45 (2.39-2.51)  1.84 (1.79-1.89)  1.52 (1.48-1.56)  1.26 (1.23-1.29)  1.00 | 0.91(0.89-0.93)  0.94(0.92-0.96)  0.94(0.91-0.96)  0.95(0.93-0.98)  1.00 | 1.05(1.02-1.07)  1.01(0.98-1.03)  1.00(0.98-1.02)  1.01(0.98-1.03)  1.00 | 0.80(0.77-0.83)  0.84(0.81-0.87)  0.87(0.84-0.90)  0.90(0.87-0.93)  1.00 |

†Covariates mutually adjusted

## Supplementary Table 7: Descriptive table for people with depression, defined as any hospital admission record in primary analyses and psychiatric hospital admission record only in sensitivity analyses.

|  | Depression defined as acute or psychiatric hospital admission record - primary analysis | Depression defined as psychiatric admission record only -sensitivity analysis |
| --- | --- | --- |
| Number with depression with and without cancer | 128,564 | 92,437 |
| Male | 46,337 | 36,998 |
| Female | 82,227 | 55,439 |
|  |  |  |
| Number with depression with cancer | 12,802 | 12,011 |
|  |  |  |
| Mean age (SD) of those admitted with depression |  |  |
| All | 54.1 (20.0) | 46.63 (18.2) |
| Male | 52.5 (19.0) | 45.2 (17.3) |
| Female | 54.9 (20.6) | 47.6 (18.8) |
|  |  |  |
| Mean age (SD) of cancer ascertainment |  |  |
| All | 69.5 (12.7) | 67.26 (12.0) |
| Male | 70.0 (11.6) | 67.23 (11.1) |
| Female | 69.8 (13.2) | 67.28 (12.5) |

## Supplementary Table 8: Age standardized cancer incidence (rate per 1,000 person-years with 95% confidence intervals (CI)) of those with and without depression (as defined in primary and sensitivity analyses), stratified by sex and time period.

|  |  | | All cancer incidence, rate per 1,000 person-years (95% CI) | | |
| --- | --- | --- | --- | --- | --- |
| Period (years) | **Those with depression defined as acute or psychiatric hospital admission record - primary analysis** | **Those without depression (acute or psychiatric hospital admission records subtracted from general population)** | | **Those with depression defined as psychiatric admission record only -sensitivity analysis** | **Those without depression (psychiatric hospital admissions records subtracted from general population)** |
| Female |  |  | |  |  |
| 1991-1994 | 13.19 (11.90 -14.66) | 10.41 (10.32-10.50) | | 14.15 (13.01-15.69) | 10.40 (10.31-10.49) |
| 1995-1999 | 12.69 (11.73 -13.73) | 10.86 (10.77-10.94) | | 13.11 (12.28-14.03) | 10.85 (10.77-10.93) |
| 2000-2004 | 13.15 (12.36 -14.01) | 10.72 (10.64-10.80) | | 14.30 (13.50-15.16) | 10.70 (10.62-10.78) |
| 2005-2009 | 13.60 (12.89 -14.34) | 11.11 (11.03-11.19) | | 13.68 (12.94-14.47) | 11.12 (11.04-11.20) |
| 2010-2014 | 14.11 (13.46 -14.78) | 11.25 (11.17-11.33) | | 14.29 (13.56-15.06) | 11.26 (11.18-11.34) |
| 2015-2019 | 12.82 (12.26 -13.41) | 10.81 (10.73-10.89) | | 13.55 (12.86-14.28) | 10.81 (10.74-10.89) |
|  |  |  | |  |  |
| Male |  |  | |  |  |
| 1991-1994 | 20.75 (18.09 -24.20) | 14.81 (14.67-14.96) | | 21.71 (19.02-38.34) | 14.69 (14.55-14.83) |
| 1995-1999 | 16.64 (15.08 -18.46) | 14.79 (14.67-14.91) | | 21.44 (19.42-24.07) | 14.76 (14.64-14.88) |
| 2000-2004 | 18.30 (16.89 -19.83) | 14.35 (14.24-14.47) | | 18.83 (17.33-20.7) | 14.35 (14.23-14.46) |
| 2005-2009 | 17.84 (16.65 -19.12) | 14.42 (14.31-14.53) | | 17.94 (16.62-19.43) | 14.42 (14.31-14.53) |
| 2010-2014 | 17.05 (16.01 -18.15) | 14.23 (14.13-14.34) | | 16.39 (15.26-17.65) | 14.25 (14.14-14.35) |
| 2015-2019 | 15.44 (14.55 -16.41) | 13.28 (13.19-13.37) | | 15.95 (14.92-17.07) | 13.28 (13.19-13.37) |

## Supplementary Table 9: Sex-stratified relative risks (95% CI) for cancer in people with versus without depression in those aged 70-74 years in Scotland in 2015 and 2019, defining depression as in primary and sensitivity analyses

| Area based deprivation | Relative Risk with 95% CI in 2015 | | Relative Risk with 95% CI in 2019 | |
| --- | --- | --- | --- | --- |
|  | Aged 70-74 years with depression defined as acute or psychiatric hospital admission record - **primary analysis** | Aged 70-74 years with depression defined as psychiatric admission record only - **sensitivity analysis** | Aged 70-74 years with depression defined as acute or psychiatric hospital admission record - **primary analysis** | Aged 70-74 years with depression defined as psychiatric admission record only - **sensitivity analysis** |
| Females |  |  |  |  |
| 1 (most deprived) | 1.25 (1.16-1.34) | 1.37 (1.27-1.47) | 1.25 (1.14-1.36) | 1.36 (1.24-1.48) |
| 2 | 1.30 (1.21-1.40) | 1.37 (1.27-1.47) | 1.30 (1.18-1.42) | 1.35 (1.24-1.48) |
| 3 | 1.32 (1.23-1.42) | 1.35 (1.25-1.40) | 1.32 (1.20-1.44) | 1.34 (1.22-1.46) |
| 4 | 1.20 (1.11-1.29) | 1.29 (1.20-1.40) | 1.19 (1.08-1.31) | 1.28 (1.17-1.40) |
| 5 (least deprived) | 1.27 (1.18-1.38) | 1.27 (1.17-1.38) | 1.27 (1.15-1.40) | 1.36 (1.15-1.38) |
|  |  |  |  |  |
| Males |  |  |  |  |
| 1 (most deprived) | 1.23 (1.15-1.33) | 1.33 (1.24-1.43) | 1.22 (1.12-1.32) | 1.32 (1.21-1.44) |
| 2 | 1.28 (1.19-1.39) | 1.33 (1.23-1.43) | 1.27 (1.16-1.38) | 1.31 (1.20-1.44) |
| 3 | 1.30 (1.21-1.41) | 1.31 (1.21-1.42) | 1.28 (1.18-1.40) | 1.30 (1.18-1.42) |
| 4 | 1.18 (1.08-1.28) | 1.26 (1.16-1.36) | 1.17 (1.07-1.27) | 1.24 (1.13-1.36) |
| 5 (least deprived) | 1.26 (1.15-1.36) | 1.24 (1.14-1.34) | 1.24 (1.13-1.30) | 1.22 (1.12-1.34) |
